# Adverse Childhood Experiences and Adolescent Mental Health Symptoms: The Role of Peer Interactions

**DOI:** 10.1007/s40653-025-00756-4

**Published:** 2025-09-10

**Authors:** Brittany Corkish, Philip J. Batterham, Kate Maston, Samantha Tang, Alison L Calear, Helen Christensen, Aliza Werner-Seidler

**Affiliations:** 1 Black Dog Institute, UNSW Sydney, Sydney, NSW Australia; 2 Centre for Mental Health Research, Australian National University, Canberra, ACT Australia; 3 School of Psychiatry, Medicine and Health, UNSW Sydney, Sydney, NSW Australia; 4 School of Psychology, UNSW, Sydney, NSW Australia

**Keywords:** Adverse childhood experiences, Adolescents, Social support, Externalizing, Internalizing

## Abstract

Adverse Childhood Experiences (ACEs) are associated with poor mental health. Evidence suggests that positive peer interactions may buffer these effects, while negative peer interactions may exacerbate this relationship. This longitudinal study assessed Australia adolescents in Year 8 at baseline (*N* = 4074, mean age 13.89 years) who reported ACEs, internalising and externalising symptoms, and peer interactions (positive and negative) between August 2019 and December 2023. Linear mixed model analyses examined the relationship between ACEs at baseline, and at mental health symptoms at 24-months, and the effects of positive and negative peer interactions. ACEs were associated with greater internalising and externalising symptoms at 24-months (*t*(3905.81) = 11.45, *p* < .001; *t*(3903.52) = 15.58, *p* < .001, respectively). No significant interaction effects were found between ACEs and positive peer social interactions for internalising or externalising symptoms (*p's* > .05). However, negative peer social interactions significantly exacerbated the relationship between ACEs and internalising and externalising symptoms over time (*t*(3912.300)= -2.86, *p* = .0.004; *t*(3906.79)=-0.200, p=.<0.001, respectively).

Adverse Childhood Experiences (ACEs) refer to events that threaten the physical, emotional, sexual and/or psychological safety of a young person and include instances of abuse and neglect (Felitti, Anda, Nordenberg, Williamson, Spitz, Edwards & Marks, 1998). In Australia, national estimates suggest approximately 62% of young people aged 16 years and over have experienced one or more ACE, with 39% experiencing multiple ACEs (Higgins, Mathews, Pacella, Scott, Finkelhor, Meinck, Erskine, Thomas, Lawrence, Haslam, Malacova & Dunne, 2023). As exposure to number of ACEs increases, mental health progressively declines (Daníelsdóttir, Aspelund, Shen, Halldorsdottir, Jakobsdóttir, Song, Lu, Kuja-Halkola, Larsson, Fall, Magnusson, Fang, Bergstedt & Valdimarsdóttir, 2024). Recent research has focused on individual factors that may mitigate the relationship between ACEs and mental health, with much of the focus on the role of social interactions. Studies have found that positive peer interactions may buffer the effects of ACEs on mental health (Yule et al., 2019), while conversely, negative peer interactions may exacerbate the impact of ACEs on mental health (Wang, Merrin, Kiefer, Jackson, Huckaby, Pascarella, Blake, Gomez & Smith, 2023; Zielinski & Bradshaw, 2006), and negatively impact the quality of peer relations (Wang et al., 2025). However, the literature is limited by having largely employed cross-sectional designs and adult samples (Struck et al., 2021). It is therefore important to investigate the role of social support in this relationship in younger samples using longitudinal designs, which ultimately may inform targeted approaches to reduce the impact of ACEs on subsequent mental health.

Research has shown that there is a dose-response relationship between ACE exposure and mental health (Daníelsdóttir et al., 2024), with symptoms increasing in severity as exposure to ACEs increases (Gu et al., 2022; Merrick et al., 2017). A recent meta-analysis which drew from three large Australian cross-sectional national surveys reported that ACEs are responsible for 21–41% of common mental health issues (Grummitt et al., 2024). These findings suggest that preventing ACEs in Australia has the potential to prevent over 1.8 million cases of mental disorders, save 66,143 years of life, and prevent 184,636 disability-adjusted life years (Grummitt et al., 2024), underscoring the urgent need for advancing our understanding of factors which prevent or influence the effects of ACEs.

Although ACE exposure is reliably associated with mental health symptoms, research has identified possible protective factors that mitigate or reduce the risk of psychopathology (Goldenson et al., 2021; Yule et al., 2019). A meta-analysis in this area found that, support received from school, peers and family was associated with reduced risk of mental health symptoms following ACE exposure (Yule et al., 2019). However, most of these included studies were cross-sectional in nature. Although there has been some more recent research showing that social and school connection reduced the risk of depression and anxiety following trauma (Gajos et al., 2022), longitudinal research is needed to establish robust evidence for directional effects.

Social connection and specifically, peer and friendship interactions become central to personal development during adolescence. Young people begin to spend increasing periods of time with peers and display a heightened sensitivity to both peer acceptance and rejection (Foulkes & Blakemore, 2016; Lam et al., 2014). This individuation allows young people to develop their own sense of self and a social identity (Orben et al., 2020; Pfeifer & Berkman, 2018). Concurrently, young people experience significant cognitive growth that increases their capacity for emotion regulation, mentalising, and interpreting social cues (Andrews et al., 2021). At this time, young people are particularly susceptible to peer influence. For example, peers can have a positive influence on academic self-efficacy (Wu et al., 2022), prosocial behaviour (Osei, 2021; Van Hoorn et al., 2016) and can reduce risk of depressive symptoms (Gariépy et al., 2016). Conversely, negative peer interactions including peer victimisation and rejection, are significant stressors and can contribute to maladaptive emotion regulation, potentially exacerbating existing emotional difficulties and leading to other negative outcomes, such as loneliness, depressive symptoms, and increased risk-taking behaviour (Schwartz-Mette et al., 2020; Van Hoorn et al., 2017).

Despite the developmental significance of peer support during adolescence, research into the effects of ACEs has predominately focused on adults and has depended on retrospective accounts of their childhood experiences (Sahle, Reavley, Li, Morgan, Yap, Reupert & Jorm, 2022; Struck et al., 2021). Therefore, the impact of peer interactions on the relationship between ACEs and internalising and externalising symptoms has not yet been explored within a large, longitudinal, representative sample of young people. Previous research has predominately used cross-sectional methodologies focused on adult samples (Struck et al., 2021), and utilised diagnostic-specific screening measures which do not capture broader symptoms of mental illness (Goodman et al., 2010). Longitudinal designs allow for establishment of directionality, and to assess the development of mental health symptoms over time. To address these research gaps, the objective of this study was to examine the role of social support from peers in the relationship between ACE exposure and mental health symptoms in a large representative sample of young people using a longitudinal design.

The study’s first aim was to replicate and extend previous findings by examining whether increased exposure to ACEs was longitudinally associated with mental health symptoms. Based on previous research (Bevilacqua et al., 2021), we hypothesised that greater levels of ACEs reported at baseline would predict greater levels of internalising and externalising symptoms over time (24-month follow-up). The second aim was to examine the effect of peer social support at baseline on internalising and externalising symptoms at 24-months. Based on previous research (Wang et al., 2021; Yule et al., 2019; Zielinski & Bradshaw, 2006), we hypothesised that positive social interactions from peers would be associated with less severe internalising and externalising symptoms, while negative social interactions with peers would be associated with more severe internalising and externalising symptoms. Finally, our third aim was to examine whether positive peer social support buffered the negative mental health effects of ACE exposure on internalising and externalising symptoms at 24-months. Our hypothesis was that there would be a buffering effect of peer positive social interactions on this relationship, and that negative peer social interactions would exacerbate the relationship between ACEs and mental health symptoms (Herd & Kim-Spoon, 2021; Yule et al., 2019).

## Method

### Study Design

Data was collected as part of a longitudinal cohort study (the Future Proofing Study) investigating risk and protective factors for adolescent mental health, conducted in Australia (Werner-Seidler, Huckvale, Larsen, Calear, Maston, Johnston, Torok, O’Dea, Batterham, Schweizer, Skinner, Steinbeck, Ratcliffe, Oei, Patton, Wong, Beames, Wong, Lingam et al., 2020; Werner-Seidler, Maston, Calear, Batterham, Larsen, Torok, O’Dea, Huckvale, Beames, Brown, Fujimoto, Bartholomew, Bal, Schweizer, Skinner, Steinbeck, Ratcliffe, Oei, Venkatesh et al., 2023). This study received ethical approval from the University of New South Wales Human Research Ethics Committee (HC180836) and is registered with the Australian New Zealand Clinical Trials Registry (ANZCTRN12619000855123). Data analysed within the current paper was collected between August 2019 and December 2023 and includes both baseline assessment and 24-month follow-up assessment.

### Participants

This study recruited participants, enrolled in year 8 from 132 schools within Australia. Of these, 49.7% were government schools and 50.3% were non-government schools, with a median ICSEA of 1055, slightly above the national median of 1000 (Werner-Seidler et al., 2023). Schools across Australia were approached to participate in the study and then recruited in three intakes over three years. After schools agreed to participate, parents and students were informed about the study via email or newsletters, with study information provided electronically. Both parental and student consent were required for participation. Consent forms were distributed electronically. The consent rate (of those eligible) into the study was approximately 31%.

### Measures

Demographic characteristics for participants included age, gender, sexuality, culturally and linguistically diverse (CALD) status, country of birth, and socio-economic status (SES); measured using a school-level Index of Community Socio-Educational Advantage (ICSEA) where 1000 is the median score, with scores below indicating reduced socio-educational advantage, and scores above 1000 indicated increased socio-educational advantage (Australian curriculum, assessment and reporting authority, 2020). Further details are provided in Table 1.

**Table 1:** Participant characteristics

Variables	*N* = 4074
Age at baseline, mean (SD)	13.89 (0.57)
**Gender**	
Female, n (%)	2060 (50.6%)
Male, n (%)	1824 (44.8%)
Non-binary, n (%)	79 (1.9%)
A different gender identity, n (%)	45 (1.1%)
Prefer not to say, n (%)	66 (1.6%)
**Sexuality**	
Heterosexual, n (%)	2860 (72.6%)
Sexuality diverse, n (%)	506 (12.9%)
Not sure/Questioning, n (%)	366 (9.3%)
Prefer not to say, n (%)	184 (4.7%)
Undetermined, n (%)	21 (0.5%)
Unreported, n	137
**Index of Community Socio-Educational Advantage (ICSEA)**	
< 1000, n (%)	1088 (26.7%)
> 1001, n (%)	2986 (73.3%)
**Aboriginal and Torres Strait Islanders**	
Not Aboriginal or Torres Strait Islander, n (%)	3676 (93.5%)
Aboriginal or Torres Strait Islander, n (%)	168 (4.3%)
Prefer not to say, n (%)	89 (2.3%)
**Culturally and Linguistically Diverse (CALD)**	
English	3797 (93.2%)
Language other than English spoken at home	276 (6.8%)
**Birth Country**	
Born in Australia	3717 (91.2%)
Born in a country other than Australia	357 (8.8%)
**ACEs**	
ACE, mean (SD)	1.07 (1.32)
Participants who experienced 1 or more, n (%)	2270 (56.5%)
**Social Support**	
Positive social interactions, mean (SD)	3.26 (0.72)
Negative social interactions, mean (SD)	1.99 (0.69)
**Symptoms (SDQ)**	
Internalizing symptoms, mean (SD)	5.90 (3.93)
Externalizing symptoms, mean (SD)	6.81 (4.00)

### ACEs

The Trauma Behavioural Risk Factor Surveillance System—Adverse Childhood Experience Module (BRFSS-ACE; Centers for Disease Control and Prevention, 2009) assessed lifetime exposure to ACEs at baseline, via self-report. The ACEs assessed included: household mental illness; household alcohol use; household illegal street drug use or prescriptive drug abuse; household incarceration; parental separation or divorce; and being sworn at, insulted or put down by a parent or adult in the home. An additional two items assessed out-of-home care arrangements, and feelings of endangerment or risk of physical harm. The current study used a modified version which did not assess physical and sexual abuse. This adaptation was made because assessments occurred at school and appropriate follow-up support could not be guaranteed. The module was scored by summing the number of endorsed events to form an ACE score of 0–8, with higher scores indicating higher exposure to adverse experiences. The BRFSS-ACE is a valid measure of early life adversity, can be mapped onto a single factor, and has alphas that range from 0.61 to 0.80 (Ford, Merrick, Parks, Breiding, Gilbert, Edwards, Dhingra, Barile & Thompson, 2014).

### Social Support

The Schuster Social Support Scale (SSSS; Schuster et al., 1990) is a 15-item self-report measure administered at baseline that assesses social interactions. This study utilised the ‘friends’ subscale only. The items within the SSSS are rated on a 4-point Likert scale from 1 (‘Never’) through to 4 (‘Often’). Items measured either positive or negative interactions, with relevant item scores averaged to produce a total positive interaction score (range = 1–4) and a total negative interaction score (range = 1–4). Higher scores on the positive dimension indicate more supportive interactions and higher scores on the negative dimension indicate more negative interactions. Internal consistency (Cronbach’s alpha) for the positive peer interaction scale was .80, and .76 for the negative peer interaction scale.

### Internalising and Externalising Symptoms

The Strengths and Difficulties Questionnaire (SDQ; Goodman, 1997) assesses young people’s emotion and behaviour via self-report. The SDQ was administered at 24 months and includes 5 subscales; emotional symptoms, conduct problems, hyperactivity/inattention, peer relationship problems and prosocial behavior. Responses are rated on a 3-point Likert scale that include: 0 (Not true), 1 (Somewhat true), or 2 (Certainly true). Total scores for Internalising (emotional symptoms and peer relationship problems) and Externalising (conduct and hyperactivity problems) symptoms were used in this study, with scores for each subscale ranging from 0 to 20 (Goodman et al., 2010). Taking this approach allows for the assessment of sub-threshold symptoms and is recommended for studies involving the general population (Goodman et al., 2010). The SDQ has demonstrated good internal consistency (Goodman, 2001; Muris et al., 2004). Internal consistency (Cronbach’s alpha) for the externalising scale is 0.40, and 0.68 for the internalising scale.

### Statistical Analysis

Participant characteristics were reported using descriptive statistics. Linear mixed model analyses were conducted to examine the effects of ACE exposure reported at baseline on internalising and externalising symptoms at 24-month follow up, and the possible role of social support (reported at baseline) in this relationship, adjusted for covariates. A random intercept for school was included in all models described to account for the cluster sampling used in the broader Future Proofing Study. An α value of 0.01 was used for statistical significance to account for multiple comparisons, and all analyses were conducted in SPSS v26.

### Model Specifications

To examine the fixed effects of ACEs on internalising and externalising symptoms while accounting for potential covariates, five potential demographic covariates (gender, sexuality, socio-economic status measured via the ICSEA, CALD, and country of birth) were entered into the model as fixed effects (Model 1). The variables that were not associated with symptoms on the omnibus test were removed from further models (CALD and country of birth). In Model 2, the three remaining significant covariates (gender, sexuality, ICSEA) were then entered into the model as fixed effects, together with baseline positive and negative social support as fixed effects, and baseline mental health symptoms (Model 2). Interaction terms were then added into this model between positive social interactions and ACEs, and negative social interactions and ACEs, to assess for the influence of positive or negative social interactions on the relationship between ACEs and symptoms (Model 3). Variables in the interaction terms were standardised.

## Results

### Descriptive Statistics

Detailed baseline participant characteristics are summarised in Table 1. Overall, there were 4074 (mean age 13.89 years at baseline, 50.6% female, 72.6% heterosexual) participants included in the final sample for this study. Participants were required to have data available at 24-month follow-up on the outcome measure (symptoms) for inclusion in the current study. At follow up participants were aged 15.83 years. There was missing data on the sexuality and social support variables (approx. 3%), and this was assumed to be missing at random. On average, participants reported exposure to 1.07 ACE.

### Primary Analyses

Results from the linear mixed-effects models are presented in Table 2. Correlation coefficients are presented in Tables 3, 4 and 5 in the supplementary materials.

**Table 2:** Results of fixed effects of ACE variables and covariates for outcomes variables

	Internalising symptoms				Externalising symptoms			
	Est.	Test	df	*p*	Est.	Test	df	*p*
**Model 1: Main effects**								
ACE total	0.49	11.45	3905.81	< 0.001*	0.69	15.58	3903.52	< 0.001*
Gender^a^								
Male	-3.16	-11.05	3890.83	< 0.001*	-1.88	-6.36	3889.40	< 0.001*
Female	− 0.65	-2.34	3914.82	0.020	− 0.91	-3.14	3913.59	0.002*
Gender Diverse	0.00	0.00	0.00		0.00	0.00	0.00	
LGBTQA + identity^b^								
Heterosexual	-1.18	-8.90	3897.20	< 0.001*	− 0.41	-3.018	3894.20	0.003*
Sexuality Diverse	0.00	0.00	0.00		0.00	0.00	0.00	
Index of Community Socio-Educational Advantage (ICSEA)								
≤ 1000	0.78	5.018	122.69	< 0.001*	0.16	1.03	145.89	0.306
≥ 1001	0.00	0.00	0.00		0.00	0.00	0.00	
Culturally and Linguistically Diverse (CALD)								
English	0.19	0.80	3238.22	0.422	− 0.35	-1.44	3269.81	0.149
Language other than English spoken at home	0.00	0.00			0.00	0.00	0.00	
Country of birth Born in Australia	− 0.30	-1.49	3901.56	0.136	− 0.21	− 0.98	3899.40	0.325
Born in a country other than Australia	0.00	0.00	0.00		0.00	0.00	0.00	
**Model 2: Main effects with baseline symptoms and social interactions**								
ACE total	0.08	1.91	3903.93	0.056	0.09	2.22	3914.79	0.027
Gender^a^								
Male	-1.27	-4.95	3841.93	< 0.001*	− 0.59	-2.34	3898.91	0.019
Female	0.19	0.79	3895.99	0.430	0.03	0.19	3914.48	0.906
Gender Diverse	0.00	0.00	0.00		0.00	0.00		
LGBTQA + identity^b^								
Heterosexual	− 0.45	-3.78	3842.17	< 0.001*	− 0.01	− 0.09	3901.50	0.932
Sexuality Diverse	0.00	0.00	0.00		0.00	0.00	0.00	
Index of Community Socio-Educational Advantage (ICSEA)								
≤ 1000	0.42	3.37	134.32	< 0.001*	0.09	0.69	150.51	0.490
≥ 1001	0.00	0.00	0.00		0.00	0.00		
Positive social support	0.05	0.67	3912.14	0.504	− 0.07	− 0.97	3914.58	0.332
Negative social support	0.08	1.09	3911.97	0.277	0.09	1.17	3913.97	0.240
Baseline symptoms	0.50	31.61	3910.71	< 0.001*	0.54	38.06	3913.68	< 0.001*
**Model 3: ACE×Positive social support interaction**								
ACE total	− 0.02	− 0.14	3913.88	0.882	− 0.15	-1.04	3910.75	0.300
Gender^a^								
Male	-1.27	-4.93	3839.54	< 0.001*	− 0.57	-2.28	3898.18	0.023
Female	0.20	0.81	3895.33	0.416	0.04	0.18	3913.60	0.858
Gender Diverse	0.00	0.00	0.00		0.00	0.00	0.00	
LGBTQA + identity^b^								
Heterosexual	− 0.44	-3.76	3840.62	< 0.001*	− 0.01	− 0.04	3901.25	0.967
Sexuality diverse	0.00	0.00	0.00		0.00	0.00	0.00	
Index of Community Socio-Educational Advantage (ICSEA)								
≤ 1000	0.41	3.35	134.46	< 0.001*	0.09	0.66	150.24	0.510
≥ 1001	0.00	0.00	0.00		0.00	0.00	0.00	
Positive social support	0.01	0.09	3911.25	0.929	− 0.17	-1.84	3913.45	0.065
Negative social support	0.08	1.07	3910.57	0.284	0.08	1.14	3913.03	0.256
Baseline symptoms	0.50	31.60	3909.82	< 0.001*	0.54	38.04	3912.31	< 0.001*
ACE×Positive social support	0.03	0.70	3914	0.484	0.08	1.729	3911.03	0.084
**Model 4: ACE×Negative social support interaction**								
ACE total	0.38	3.35	3913.35	< 0.001*	0.52	4.55	3906.86	< 0.001*
Gender^a^								
Male	-1.26	-4.91	3840.62	< 0.001*	− 0.57	-2.27	3897.73	0.023
Female	0.22	0.91	3894.98	0.366	0.07	0.28	3913.40	0.779
Gender Diverse	0.00	0.00	0.00		0.00	0.00	0.00	
LGBTQA + identity^b^								
Heterosexual	− 0.44	-3.71	3842.62	< 0.001*	0.00	0.02	3900.89	0.981
Sexuality Diverse	0.00	0.00	0.00		0.00	0.00	0.00	
Index of Community Socio-Educational Advantage (ICSEA)								
≤ 1000	0.43	3.44	131.99	< 0.001*	0.11	0.79	149.69	0.431
≥ 1001	0.00	0.00	0.00		0.00	0.00	0.00	
Positive social support	0.04	0.59	3911.06	0.558	− 0.08	-1.08	3913.57	0.281
Negative social support	0.25	2.61	3910.25	0.009	0.32	3.40	3912.28	< 0.001*
Baseline symptoms	0.50	31.51	3909.74	< 0.001*	0.54	37.91	3912.35	< 0.001*
ACE×Negative social support	− 0.14	-2.86	3912.30	0.004	− 0.20	-4.03	3906.79	< 0.001*

*. Correlation is significant at the 0.01 Level

^a^For analyses including the gender variable, participants were categorised into a female, male and a diversity category (nonbinary, a different gender identity and prefer not to say) because of small cell sizes

^b^For sexuality analyses, participants were categorised into a heterosexual, and a diversity category (all other options) because of small cell sizes

**Table 3:** Means, standard deviations (SD), and correlation between social support, trauma, distress and symptoms at baseline

Variables	Mean (SD)	ACE’s	Positive peer interactions	Negative peer interactions	SDQ Internalizing	SDQ Externalizing
ACEs	1.18 (1.42)	-				
Positive peer interactions	3.24 (0.74)	− 0.200*	-			
Negative peer interactions	2.00 (0.71)	0.185*	− 0.240*	-		
SDQ Internalizing	5.90 (3.93)	0.343*	− 0.349*	0.288*	-	
SDQ Externalizing	6.81 (4.00)	0.402*	− 0.262*	0.294*	0.525*	-

*. Correlation is significant at the 0.01 Level

**Table 4:** Means, standard deviations (SD), and correlation between social support, trauma, distress and symptoms at 12 months

Variables	Mean (SD)	ACE’s	Positive peer interactions	Negative peer interactions	SDQ Internalizing	SDQ Externalizing
ACEs	1.18 (1.42)	-				
Positive peer interactions	3.24 (0.74)	− 0.200*	-			
Negative peer interactions	2.00 (0.71)	0.185*	− 0.240*	-		
SDQ Internalizing	5.82 (3.89)	0.240*	− 0.210*	0.177*	-	
SDQ Externalizing	6.73 (3.88)	0.309*	− 0.199*	0.208*	0.497*	-

*. Correlation is significant at the 0.01 Level

**Table 5:** Means, standard deviations (SD), and correlation between social support, trauma, distress and symptoms at 24 months

Variables	Mean (SD)	ACE’s	Positive peer interactions	Negative peer interactions	SDQ Internalizing	SDQ Externalizing
ACEs	1.18 (1.42)	-				
Positive peer interactions	3.24 (0.74)	− 0.200*	-			
Negative peer interactions	2.00 (0.71)	0.185*	− 0.240*	-		
SDQ Internalizing	5.88 (3.84)	0.231*	− 0.186*	0.157*	-	
SDQ Externalizing	6.66 (3.76)	0.273*	− 0.186*	0.191*	0.468*	-

*. Correlation is significant at the 0.01 Level

In Model 1 (base model with main effects), greater levels of ACE exposure at baseline were associated with more severe internalising symptoms (*t*(3905.81) = 11.45, *p* < .001), and more severe externalising symptoms (*t*(3903.52) = 15.58, *p* < .001) at 24-months. In Model 2, when baseline internalising and externalising symptoms were entered into the model, neither positive nor negative social interactions were significantly associated with internalising symptoms (*t*(3912.14) = 0.67, *p* = .504; *t*(3911.97) = 1.09, *p* = .277), or externalising symptoms (*t*(3914.58)=-0.97, *p* = .332; *t*(3913.97) = 1.17, *p* = .240) at 24-months. However, the association between ACE exposure with baseline internalising and externalising symptoms remained significant.

When an interaction term between ACE exposure and positive social interactions was introduced in Model 3, fixed effects of positive and negative social interactions remained non-significant (*p* > .05), and there was no interaction effect between ACEs and positive social interactions on subsequent internalising symptoms (*t*(3914) = 0.70, *p* = .484), nor externalising symptoms (*t(*3911.03) = 1.73,*p* = .084).

When an interaction term between ACE exposure and negative social interactions was introduced in Model 3, there remained no significant effect of positive social interactions on internalising and externalising symptoms. However, negative social interactions were significantly associated with both symptom domains (internalising: *t*(3910.25) = 2.61, *p* = .009, externalising: *t*(3912.28) = 3.40, *p* < .001). There were also significant interactions between negative social interactions and ACEs for internalising symptoms (*t*(3912.30)= -2.86, *p* = .0.004), and externalising symptoms (*t*(3906.79) = − 0.20, *p*=.<0.001). This suggests that participants who experienced a greater number of ACEs, together with more negative social interactions, were more likely to have greater internalising and externalising symptoms 24-months later. There was no evidence for a buffering effect on the relationship between ACEs and symptoms by positive social interactions.

## Discussion

This study examined the longitudinal relationship between ACEs, mental health symptoms and social interactions with peers. As hypothesised, we found greater levels of ACEs reported at baseline predicted more severe internalising and externalising symptoms 24 months later. We also found that greater experiences of negative interactions were associated with internalising and externalising symptoms 24 months later. Contrary to our third hypothesis, we found that positive social interactions did not provide any buffer or protection against the development of mental health symptoms following ACE exposure. However, experiences of negative peer interactions were found to exacerbate mental health symptoms following ACE exposure. Within each model, baseline mental health symptoms were a strong predictor of subsequent symptoms at 24-months, underscoring the need for early intervention. Considered together, our results replicate the finding that ACE exposure is associated with poorer mental health, and that this effect persists over time, and is exacerbated by negative peer interactions (Bevilacqua et al., 2021; Daníelsdóttir et al., 2024; Herd & Kim-Spoon, 2021; Merrick et al., 2017).

The relationship between ACEs and mental health symptoms is consistent with previous research (Bevilacqua et al., 2021; Daníelsdóttir et al., 2024; Herd & Kim-Spoon, 2021). However, contrary to findings from a meta-analysis by Yule et al. (2019), positive social interactions did not emerge as a protective factor following ACE exposure. This result may have occurred for a number of reasons. Firstly, the outcomes assessed within this study were internalising and externalising symptoms, whereas the studies reviewed by Yule et al. (2019) examined adaptive functioning within studies that included both children and adolescent populations. Furthermore, within this meta-analysis effect sizes within cross sectional studies were stronger, suggesting that concurrent peer support may be a more protective buffer rather than peer support as a protective factor for subsequent mental health.

Past studies that have found a buffering effect of positive social interactions did not focus on adolescents still residing in the family home and instead utilized retrospective self-reports during adulthood (Brinker & Cheruvu, 2017; Sperry & Widom, 2013). Positive peer interactions may help buffer against the effects of ACEs like sexual and physical abuse, particularly when the trauma is reported retrospectively, suggesting that increased time since the abuse may play a role. Although speculative, it may be the case that peer interactions are less effective in buffering against ACEs related to general family dysfunction and emotional abuse, especially when young people still reside within the family home, and the abuse is potentially ongoing.

Early relational experiences within the home environment are crucial as they provide a blueprint for future relationships (Wang et al., 2023). Many of the ACEs assessed were likely to have occurred in the home environment (e.g., household drug and alcohol use/abuse) and although speculative, it is possible that many young people within this study may have struggled to develop adequate support networks in the absence of a foundation provided by secure family relationships, precluding a buffering effect of positive social interactions on the relationship between ACEs and mental health symptoms. The negative correlation between ACEs and positive interactions may be indicative of this. Other contributing factors that may have influenced the absence of a protective effect of positive social interactions include the participants’ age at the onset of ACEs and the severity of these experiences. The way ACEs were measured did not allow participants to indicate the timing, details, location, severity, or frequency of these experiences. Experiencing a higher number of ACEs at a younger age is known to contribute to greater levels of mental distress (Merrick et al., 2017). Accordingly, this may have had a greater influence on participants abilities to form meaningful relationships, and may have contributed to these findings.

Consistent with our findings, negative social interactions, or peer victimisation have previously been shown to exacerbate symptoms of mental ill health (Wang et al., 2023). Research by Burke et al. (2017) found that the relationship between negative social interactions and depressive symptoms are bidirectional, exacerbating each other over time. As outlined above, it is possible that the adolescents who were not provided sufficient social support to develop a relational framework may lack the skills to develop supportive social relationships, interpret and understand social cues, and may respond with maladaptive social responses such as aggressive or withdrawal behaviours (Wang et al., 2023). These challenges are likely to further isolate these young people, contributing to both mental ill health, and greater experiences of negative peer interactions. There is some research that has found benefit from providing intervention that aims to increase social connection and school belonging (Allen et al., 2022; Raniti et al., 2022). These results have been promising and could potentially address negative peer interactions, breaking the aforementioned reinforcement schedule. However, as positive social interaction did not provide protection against the negative consequences of exposure to ACEs, this solution would be insufficient alone. Due to the potential for the consequences of ACE exposure to be severe, pervasive, and long term, it is essential to try and reduce the rate of ACEs through direct prevention activities.

Preventing ACEs requires a unified, multi-dimensional approach that addresses immediate and systemic factors by targeting at-risk populations and addressing modifiable risk factors (Salter & Gore, 2020). The model proposed by Salter and Gore (2020) suggests interventions should occur at the primary, secondary and tertiary levels. Traditionally, interventions have centred on tertiary prevention, which involves supporting survivors of abuse and neglect via provision psychological support. However, directing efforts to secondary prevention by addressing those at increased risk of exposure or perpetration, as well as primary prevention approaches through community initiatives is crucial and may reduce the likelihood of young people experiencing ACEs. For example, in addition to individual psychological interventions for young people at risk of mental ill health, early parenting interventions, such as Triple P and Circle of Security provide parents with improved parenting skills and stress management skills that offer promise for both prevention and intervention of ACEs (Sanders et al., 2014; Yaholkoski et al., 2016).

While this study provides important information on the relationships between ACEs, social interactions and mental health symptoms, it also contains several limitations that warrant mention. First, participants were administered a modified version of the BRFSS-ACE that did not assess experiences of physical or sexual abuse, or the timing, details, severity, or frequency of these experiences. This was due to resource limitations in the scope of the large population-based study that precluded appropriate follow-up at the time of assessment within schools. Not including assessment of physical or sexual abuse means that two of the most common forms of adverse childhood experiences (Mathews, 2023) were not captured by our survey and therefore our findings are limited in their generalisability to ACEs broadly experienced in childhood. Furthermore, there was a low consent rate, and participants who did consent completed these assessments at school, knowing results were identifiable to the research team. This may have biased the sample, and influenced rates of disclosure, although information about confidentiality of responses was nonetheless provided. Second, although a widely used and well-validated scale, the psychometric properties of the SDQ fluctuated within this sample, with poor internal consistency for the externalising subscale, limiting the validity of the findings specific to that subscale. Additionally, this study focused on the mediating role of peer interactions. Future research may benefit from increased assessment of positive and negative interactions within additional contexts, such as school or familial interactions. Finally, while this study included a largely representative sample, the socioeconomic advantage was slightly above average, and this study was not inclusive of students who had disengaged from school, or who were completing alternative education pathways.

In a large, representative, longitudinal sample, this study reported that ACEs have a significant impact on mental health symptoms, and that social support was insufficient in protecting young people from these harms. These results demonstrate the importance of preventing ACEs, in addition to providing support to those affected by ACEs. Successful prevention and early intervention measures should include primary, secondary and tertiary approaches. If implemented, these preventative measures and intervention strategies have the potential to reduce the frequency of ACEs and subsequent mental health deterioration.

## Data Availability

All reasonable requests made to the research team will be considered.
